# Supplementary material for: Synthesis of NiMoO4/NiMo@NiS Nanorods for Efficient Hydrogen Evolution Reactions in Electrocatalysts
Source: Nanomaterials (Basel). 2023 Jun 16;13(12):1871. doi: 10.3390/nano13121871 (PMC10304860; doi:10.3390/nano13121871)
Supplement: Supplementary file 1 [file nanomaterials-13-01871-s001.zip › nanomaterials-2442567-supplementary.pdf]

Supporting Information

S1. Materials and Methods

S1.1. Chemicals and reagents

Graphite flake, Nickel nitrate hexahydrate [$Ni(NO_3)_2 \cdot 6H_2O$], ammonium molybdate [$(NH_4)_6Mo_7O_{24}$ $4H_2O$]，hydrochloric acid (HCl, 37%) were purchased from Macklin Reagent Co., Ltd. Potassium hydroxide (KOH)， Thiourea (CH_4N_2S) , Sodium citrate dihydrate [$C_6H_5Na_3O_7 \cdot 2H_2O$] were purchased from aladdin Co., Ltd. Ni foam (NF) was purchased from Shanxi lizhiyuan materials Technology Co., Ltd. All chemicals were analytical grade and used without further purification.

S1.2. Synthesis of NF@NiS

To prepare the NF sample, the NF was cut into four 1 cm × 2 cm pieces and placed in a clean Petri dish for later use. During the electrochemical deposition process, the CHI 760E workstation, comprising a three-electrode system, was utilized. The working, reference, and counter electrodes were composed of NF, a saturated silver/silver chloride electrode, and a platinum wire, respectively. The electrolyte solution consisted of approximately 50 mL of 5 mM $Ni(NO_3)_2 \cdot 6H_2O$, 5 mM CH_4N_2S, and 7.5 mM $Na_3C_6H_5O_7 \cdot 2H_2O$. To achieve the desired deposition quality, the deposition voltage, time, and stirring speed were optimized to be -0.8 V, 600 s, and 300 rpm, respectively.

S2. Electrochemical HER Performance

HER (Hydrogen Evolution Reaction) is the hydrogen-producing reaction in the process of electrolysis of water, which reduces water to hydrogen gas (H_2) under electrochemical conditions in aqueous solution. Its mechanism mainly involves reactions of water molecules, electrons, cations, and protons.

The basic mechanism of HER reaction includes the following three steps:

Adsorption: water molecules are adsorbed onto the electrode surface by electrostatic or hydrogen-bonding attractions.

Reaction: when the electrode is subjected to an electrical potential, electrons are transferred from the electrode to reduce water molecules to hydroxide ions (OH^-) and one electron.

Dissociation: after the OH^- ion loses another proton, the resulting O^{2-} ion detaches from the electrode, and the remaining two hydrogen ions combine to form H_2 and are released.

The electrocatalytic hydrogen evolution reaction of the samples was tested in a three-electrode system by an electrochemical workstation (Chenhua, CHI 760E) under alkaline conditions. In which the nickel foam loaded sample was used as the working electrode, Hg/HgO as the reference electrode and graphite rod as the counter electrode, respectively. For the LSV test, the voltage window of the scan ranged from -0.8 V to -1.5 V, and the scan rate was 0.005 V/s, and 90 % IR compensation. The EIS of the samples were obtained by testing under open-circuit voltage conditions in the frequency range of 0.01 Hz to 100000 Hz. The electric double layer capacitance (C_{dl}) was calculated by measuring the CV curves in the non-Faraday interval at different sweep rates of the sample. The stability test of the samples was obtained by chronoamperometry (i-t) and after 1000 CV cycling tests. The Tafel slope was obtained by converting the LSV curves to the following equation

$$\eta = a + b \times \log J, \quad \text{(S1)}$$

where η is the overpotential, J is the current density, and b is the Tafel slope.

All tests were performed at room temperature and the measured electrochemical data were converted to standard hydrogen electrodes in 1 mol/L KOH electrolyte by the equation

$$E(RHE) = E(Hg/HgO) + 0.098 + 0.0591 \times pH \quad \text{(S2)}$$

In the process of water electrolysis for hydrogen production, E(RHE) represents the reference electrode potential, which can be measured relative to the potential of a mercury/mercury oxide electrode (Hg/HgO). On the other hand, E(Hg/HgO) refers to the potential of the mercury/mercury oxide electrode used as the reference electrode.

The term 0.098V refers to the difference between the standard electrode potential and the standard hydrogen electrode (SHE) at room temperature, also known as the zero-point shift. pH indicates the acidity or alkalinity of the solution.

S2.1. Synthesis of Pt/C/NF electrode

The Pt/C catalyst was prepared by mixing 5 mg of commercial Pt/C (20 wt%) in a 5 wt% Nafion (50 μL) solution of anhydrous ethanol (380 μL) and water (570 μL). A 5 wt% Nafion (50 μL) solution containing anhydrous ethanol (380 μL) and water (570 μL) was strictly sonicated for 30 min to ensure uniform mixing of the Pt/C suspension. A total of 400 μL Pt/C catalyst was dropped on NF (1 cm × 1 cm) to prepare Pt/C/NF electrodes.

S3. Characterizations

X-ray diffraction (XRD) analysis was performed using a Rigaku Smart Lab 9 kW high-resolution system and Cu Kα radiation, which was used to analyze the phase composition of the material with a scan range of 10-90°. The morphology and structure of the composites were recorded by scanning electron microscopy (SEM, JSM-7610F) and transmission electron microscopy (TEM, FEI Themis Z, 300 kV). X-ray photoelectron spectroscopy (XPS, Thermo ESCALAB 250Xi) was used to probe the elemental composition and valence of the catalysts.

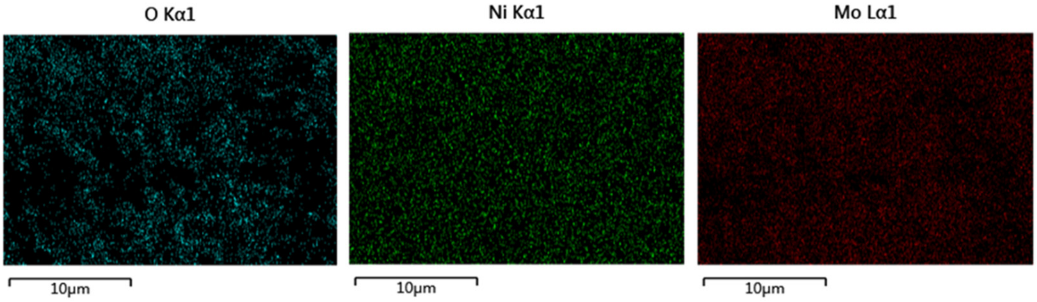

Figure S1. The EDS images of NF/$NiMoO_4$/NiMo.

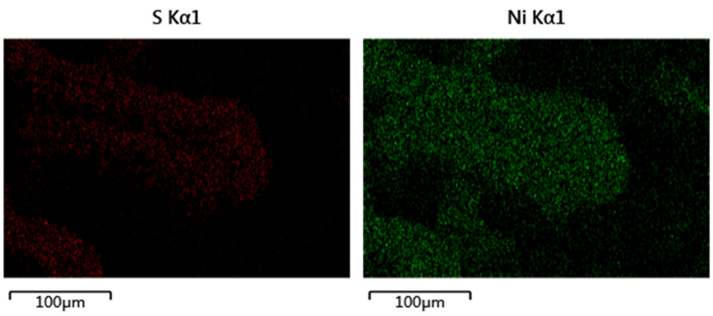

Figure S2. The EDS images of NF@NiS.

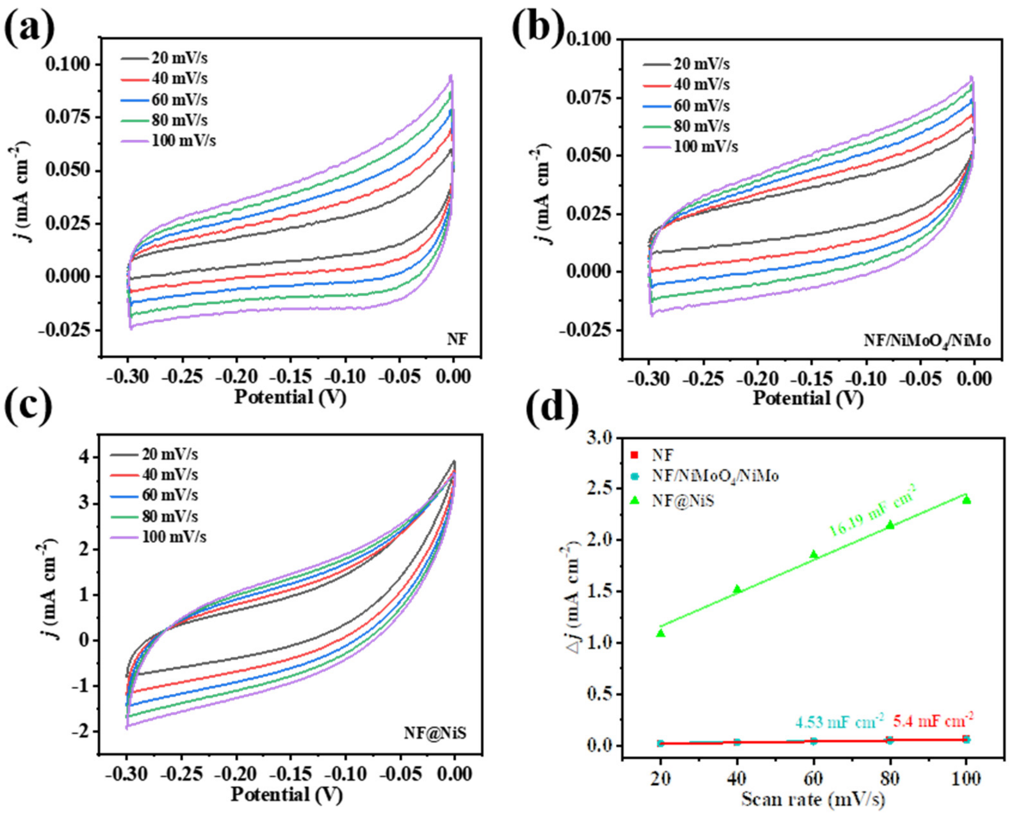

Figure S3. (a) (b)(c) Electrochemical CVs of contrast samples measured at different scan rates. (d) Electric double-layer capacitance for each sample.

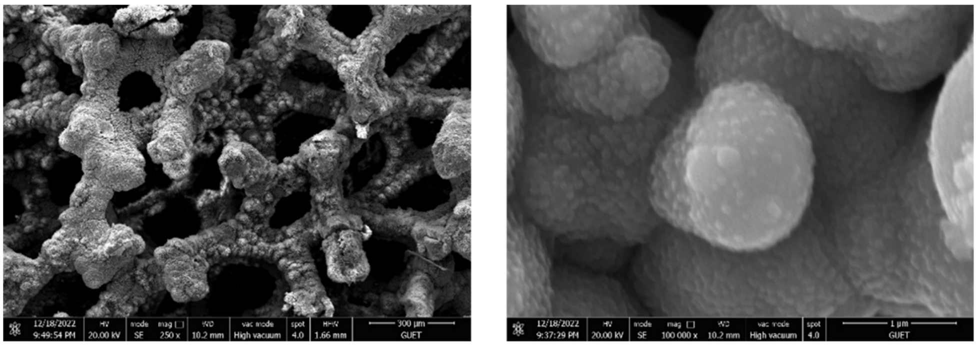

Figure S4 SEM images after reaction.

Table S1. HER electrocatalysis performance of some typical catalysts reported previously.

Sample	Electrolyte	Overpotential at 10 mA cm^{-2} (mV)	Current density (mA/cm^2)	Tafel slope (mV dec^{-1})	References
**$NiMoO_4$/NiMo@NiS**	1.0 M KOH	36	10	40.2	this work
**Co-Ni_3S_2/NF**	1.0 M KOH	148	10	68	[1]
**Ni_3S_2/NiS**	1.0 M KOH	129	10	75.5	[2]
**$Ni(OH)_2$/$CoNi_2S_4$/NF**	0.5 M H_2SO_4	124	10	84	[3]
**Zr-MOF/NiS_2**	0.5 M H_2SO_4	110	10	-	[4]
**$Cu_{7.2}S_4$@NiS_2@NiS/NF**	1.0 M KOH	87	10	43.7	[5]
**N-PECVD**	0.5 M H_2SO_4	90	10	80	[6]
**Ni-S**	1.0 M KOH	58	100	81.6	[7]
**Cu_2S/Ni_3S_2**	1.0 M KOH	50	10	-	[8]
**$NiCo_2S_4$/Ni_3S_2**	1.0 M KOH	111	10	57	[9]

Reference

[1] Sun K, Qiao F, Yang J, et al. Fluff spherical Co-Ni_3S_2/NF for enhanced hydrogen evolution[J]. Int. J. Hydrogen Energy, 2022, 47(65): 27986-27995.

[2] Chen M, Su Q, Kitiphatpiboon, N.; Zhang, J.; Feng, C.; Li, S.; Zhao, Q.; Abudula, A.; Ma, Y.; Guan, G. Heterojunction engineering of Ni_3S_2/NiS nanowire for electrochemical hydrogen evolution. *Fuel* **2023**, *331*, 1257944.

[3] Wu, Y.; Su, L.; Wang, Q.; Ren, S. In situ preparation of $Ni(OH)_2$/$CoNi_2S_4$/NF composite as efficient electrocatalyst for hydrogen evolution reaction. *Ionics* **2023**, *29*, 675–683.

[4] He, N.; Chen, X.; Fang, B.; Li, Y.; Lu, T.; Pan, L. Zr-MOF/NiS_2 hybrids on nickel foam as advanced electrocatalysts for efficient hydrogen evolution. *J. Colloid Interface Sci.* **2023**, *640*, 820–828.

[5] Gong, Y.; Lin, Y.; Yang, Z.; Jiao, F.; Li, J.; Wang, W. High-performance bifunctional flower-like Mn-doped $Cu_{7.2}S_4$@NiS_2@NiS/NF catalyst for overall water splitting. *Appl. Surface Sci.* **2019**, *476*, 840–849.

[6] Sun, M.; Wang, Z.; Gao, B.; Wang, S.; Wang, C.; Song, X.; Lin, D. Electrocatalytic hydrogen evolution properties of anionic NiS_2-$Ni(OH)_2$ nanosheets produced on the surface of nickel foam. *Int. J. Energy Res.* **2020**, *44*, 4827–4836.

[7] Wu, Y.; Lian, J.; Wang, Y.; Sun, J.; He, Z.; Gu, Z. Potentiostatic electrodeposition of self-supported NiS electrocatalyst supported on Ni foam for efficient hydrogen evolution. *Mater. Des.* **2021**, *198*, 109316.

[8] Peng, Y.; He, H. Novel heterostructure Cu_2S/Ni_3S_2 coral-like nanoarrays on Ni foam

to enhance hydrogen evolution reaction in alkaline media. *RSC Adv.* **2021**, *11*, 39493–39502.

[9] Xu, X.; Liu, Q.; Zhong, W.; Zhang, L.; Lu, Y.; Du, Y. Construction of $NiCo_2S_4/Ni_3S_2$ nanoarrays on Ni foam substrate as an enhanced electrode for hydrogen evolution reaction and supercapacitors. *Int. J. Hydrog. Energy* **2021**, *46*, 39226–39235.
